# Editorial: Early Moral Cognition and Behavior

**DOI:** 10.3389/fpsyg.2019.02013

**Published:** 2019-09-20

**Authors:** Kelsey Lucca, J. Kiley Hamlin, Jessica A. Sommerville

**Affiliations:** ^1^Department of Psychology, University of Washington, Seattle, WA, United States; ^2^Psychology Department, Arizona State University, Tempe, AZ, United States; ^3^Department of Psychology, University of British Columbia, Vancouver, BC, Canada; ^4^Department of Psychology, University of Toronto, Toronto, ON, Canada

**Keywords:** moral cognition, social cognition, infancy, early childhood, moral development

To date, research on moral cognition and behavior has focused primarily on children and adults—leaving open critical questions surrounding earlier developmental origins of morality. This special issue presents an integrative collection of pioneering research in early moral cognition and behavior that fills this gap. This work investigates a range of timely and important questions surrounding the extents of early moral cognition and behavior, demonstrating that human infants and young children have an unmatched flexibility in their thinking and acting in the moral domain: within the first several years of life, moral representations are quite robust, flexible, and complex in nature. This work also sheds light on sources of variability in moral cognition and behavior, such as interactions in the home environment, a previously understudied topic. And finally, this research provides novel insights into continuities and discontinuities in moral behavior and cognition across ages (i.e., 4 months to middle childhood), populations (i.e., children with autism, children from non-Western countries), and species (i.e., dogs). This research employs a range of methodological techniques, such as pupillometry, behavioral experiments, and large-scale survey studies that span diverse theoretical approaches, including computational modeling and constructivism. In sum, the papers in this issue stress four main themes: the extents and boundaries of early moral cognition, diverse populations and approaches, factors that moderate moral thinking and action, and new theoretical frameworks for understanding moral cognition. Here, we address each of these themes in turn, and highlight how these papers demonstrate that early moral cognition and behavior, starting in early infancy and extending into early childhood, is highly flexible, shaped in important ways by various contextual and experiential factors, and continuous across cultures and development.

The first set of papers tackle important questions regarding the extents and boundaries of early moral representations by probing infants' reasoning about the social world. Existing work has established that very young infants are sensitive to nice and mean actions: they prefer those who help over those who hinder. After infants' first birthday, they demonstrate a similar sensitivity to fairness, preferring those who behave fairly to those who don't. However, research has yet to examine these two important dimensions of morality in tandem, leaving open critical questions about the similarity in timeline of these traits, and whether infants' judgements are simply temporary evaluations, or whether they view these traits as enduring and stable behavioral dispositions. Surian et al. demonstrate that by 14 months, infants expect individuals who have previously helped (as opposed to harmed) others to be fair in future interactions, demonstrating that infants link the domains of harm, help, and fairness, and may attribute moral “traits” to others. Previous research on fairness expectations in the first year of life has yielded mixed results: some research has found that young infants expect third parties to act fairly, whereas other research has not. In a series of four experiments, Dawkins et al. resolve these disparate findings by demonstrating the precise conditions under which early fairness expectations exist: 4- and 9-month-olds are sensitive to fairness, but only when distributions are small and markedly different from each other, highlighting that although fairness expectations emerge early in development, there are also important limits to these expectations. Taborda-Osorio et al. further probe the extents of infants' sociomoral representations by asking whether infants perceive sociomoral dispositions as a deep and identity-determining features. Using an object individuation task, they find that infants interpret sociomoral actions (i.e., helping, hindering) as stable behavioral dispositions. Together, this research moves beyond past work by showing infants' fairness understanding emerges earlier than previously thought, and is flexible and cohesive across domains—highlighting that infants' judgements about the moral behavior of others are not just fleeting evaluations, but a true understanding of the behavioral dispositions that underlie the actions of others.

Historically, the field of early moral cognition and behavior has been dominated by research with children from western, educated, industrialized, rich, and democratic (WEIRD) societies, raising important questions about universality of early moral cognition and sources of variability. The research in this collection alters the course of this narrative by working with understudied populations. In an experiment examining patterns of attention to prosocial events, Hepach and Herrmann find important continuities across cultures and ages: children from 3 to 9 years in both Germany and Zambia show similar pupillary responses to helping scenarios, and process social information similarly: they are better equipped to anticipate the solution to social (compared to non-social) problems. Chernyak et al. also investigate moral cognition and behavior in Zambian children, and similarly find important cross-cultural similarities: across cultures, rates of prosociality are scaled to the cost of the action. They also identify a range of cultural factors that contribute to individual differences in moral cognition, such as parental perception of inequality. The field, prior to this special issue, has also been limited in that conclusions typically rest on experiments conducted with neurotypical children. Dunfield et al. tackle this issue by studying children diagnosed with Autism Spectrum Disorder (ASD) and find that children with ASD engage in similar levels of helping and sharing as typically developing (TD) children. However, children with ASD are less inclined to engage in prosocial behaviors when the cost of acting is high—thereby emphasizing that social cognition and social motivation combined are critical features of prosocial behavior across diverse groups.

The articles presented in this collection also diversify the field by utilizing novel approaches. Using a large-scale online survey, Hammond and Brownell map the developmental trajectory of early helping behaviors and demonstrate that children's earliest helping behaviors are driven by social engagement, praise, and fun, and that these motivations differentiate and expand across development to also include more altruistic motives. McAuliffe et al. take a comparative approach and ask whether domestic dogs, similar to human infants, form social evaluations based on third party interactions. Unlike human infants, who prefer helpers over hinderers from a very early age, dogs do not show any preference. In this way, human infants have an unmatched flexibility in their early moral cognition.

The last set of empirical papers explore a range of factors that moderate morally-relevant behavior and cognition. Prior to this collection, little was known about the relative weighting of different factors in moral-decision making at different stages of development. The papers by Van de Vondervoort et al. and Fedra and Schmidt illustrate that intentionality plays a fundamental role in early social reasoning. Van de Vondervoort et al. demonstrate that young children privilege intentions over outcomes when making moral judgements about helping and hindering agents. Fedra and Schmidt show that children's reasoning about the moral behaviors of others goes beyond actions that are intrinsically helpful and harmful, and extend to verbal actions that reveal intentions to help or harm, such as factual statements and assertions. This work highlights that the ability to inspect and appraise the moral consequences of what people say, and reason about the underlying intentional structure of actions, is an important feature of mature moral reasoning present early in life.

The papers by Lee et al. and Misch et al. demonstrate that group membership is another key factor involved in moral decision making. Their work illustrates that children treat both in-group and out-group members fairly, but will override fairness concerns in favor of group loyalty when resources are limited. Misch et al. examine how children navigate the tension between standing up for what's right and remaining loyal to a group: when the stakes for the group are low, after a minor transgression, children blow the whistle on both ingroup and outgroup transgressors—but when there stakes for the group are high, after a severe moral transgression, children are less likely to blow the whistle on an ingroup member.

Prior to this special issue, cohesive theoretical frameworks for explaining where prosocial tendencies come from and how they lead to prosocial actions were missing from the literature, making it difficult to interpret and make sense of empirical findings. The final set of papers, by Dahl and Killen and Bridgers and Gweon, offer novel theoretical perspectives on the origins of morality. In addition to providing a comprehensive and integrative definition of morality—“prescriptive norms concerning others' welfare, rights, fairness, and justice”—Dahl and Killen take a constructivist approach to interpreting the evidence on the developmental trajectory of morality, arguing that early morality is neither innate nor learned, but rather constructed through reciprocal interactions. Bridgers and Gweon also explore the question of why and how prosocial behaviors develop, with an eye toward explaining why certain behaviors tend to emerge earlier and with less prompting than others. They argue that deconstructing early prosocial behaviors into complex decision-making processes, and developing computational models that formalize these processes, can help elucidate the developmental trajectory of moral development.

Together, this collection highlights that human infants and children demonstrate an unmatched flexibility in their thinking and acting in the moral domain. This collection also points to constraints on early moral cognition and behaviors, and help elucidate the contexts in which these constraints exist—such as when group membership is at stake or when the processing load is too high. Though these papers make large strides in moving the field forward toward a more cohesive and stable representation of early morality, they also pose important questions and challenges for the field moving forward. For example, although much of the work presented here is suggestive of promising applications for fostering early moral concerns and behaviors, both the degree to which a “moral sense” is malleable and the long-term effects of early attempts at intervention remain unknown. Future work in this vein, coupled with the advancements presented in this collection, will help construct a more unified understanding of the origins and development of morality.

## Author Contributions

KL drafted the editorial. JS and JH provided critical feedback. All authors contributed equally to editing this special issue.

### Conflict of Interest

The authors declare that the research was conducted in the absence of any commercial or financial relationships that could be construed as a potential conflict of interest.

